# Editorial: Sex bias in autoimmunity: From animal models to clinical research and applications

**DOI:** 10.3389/fmed.2022.1112966

**Published:** 2022-12-13

**Authors:** George A. Robinson, Hannah Peckham, Elizabeth C. Jury, Veena Taneja, Coziana Ciurtin

**Affiliations:** ^1^Centre for Rheumatology Research, Division of Medicine, University College London, London, United Kingdom; ^2^Centre for Adolescent Rheumatology Versus Arthritis, Division of Medicine, University College London, London, United Kingdom; ^3^Department of Immunology, The Mayo Clinic, Rochester, MN, United States

**Keywords:** autoimmunity, sex differences, translational research, rheumatoid arthritis, gender dysphoria

Considerable progress in generating and reporting scientific data disaggregated by sex has been achieved in the last decades since the Food and Drug Administration (FDA) recommendation in 1998 to provide the age, sex, and ethnicity of all participants in clinical trials. The increasing focus on recognizing sex differences in immune responses is particularly relevant for autoimmune diseases, such as systemic lupus erythematosus (SLE), Sjögren's syndrome (SS) and rheumatoid arthritis (RA) which typically affect women more than men. We are beginning to understand key differences in the immune system that may help to explain sexual dimorphisms in autoimmune disease risk and manifestations, as well as prognosis, complications, and response to treatment. There is also an increased interest in investigating how changes in human physiology (e.g., during pregnancy, lactation, menopause) and other sex and gender differences, pertaining to socio-economic and educational factors, as well as access to care and health-related behaviors can affect the immune system, advocating the need for sex and gender inclusive research. Unique gender-diverse human cohorts and animal models are aiding our understanding of how sex and gender can influence the immune system and various metabolic pathways relevant to autoimmunity (Figure 1).

**Figure 1:**
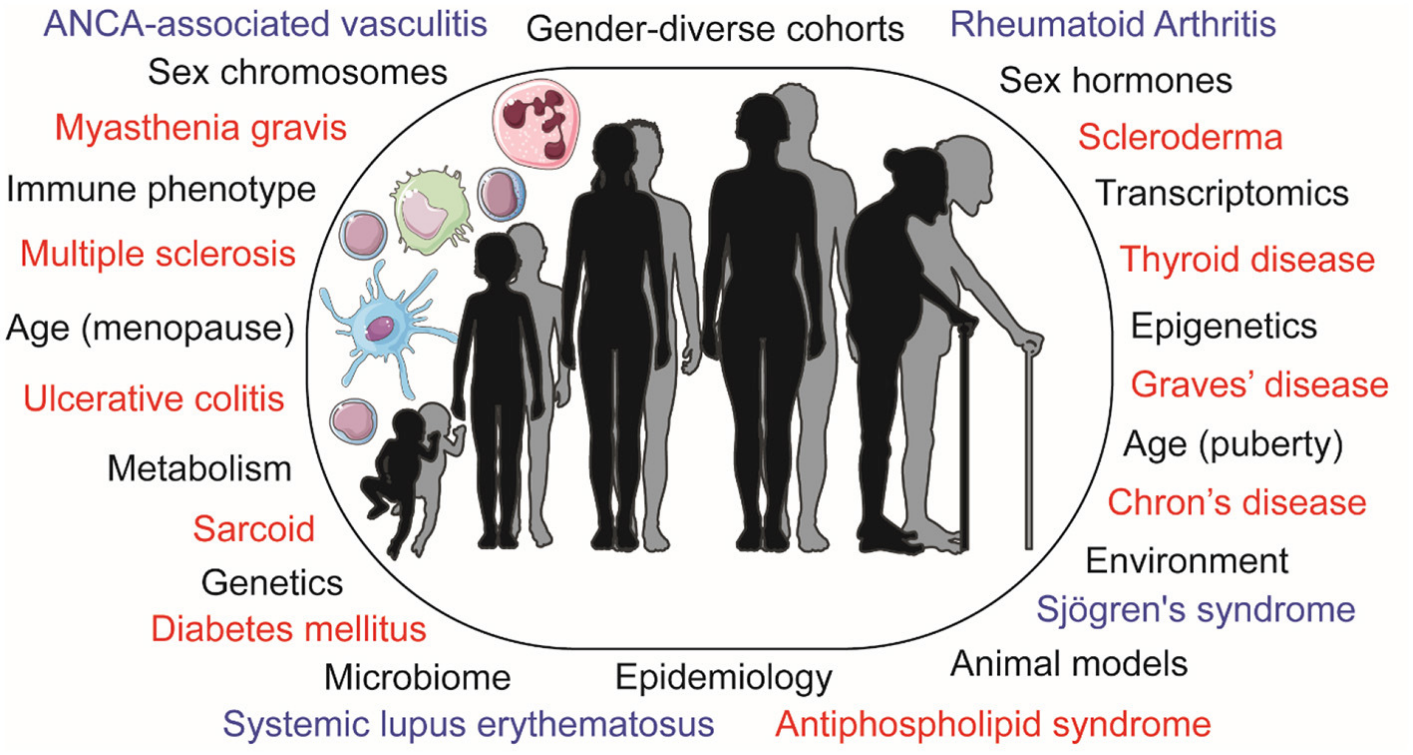
Schematic representation of the differences observed in immunology and autoimmune disease risk by sex and age, displaying pathophysiological influences and research methods used to explore these in black. Autoimmune diseases that present with a sex bias are displayed in blue (for those covered in this article) or red. ANCA, anti-neutrophil cytoplasmic antibodies.

There is convincing literature evidence that sex hormones differentially regulate the phenotype of various immune cells *in vitro* with implications for the pathogenesis of SLE, a disease with a profound female bias (Kim et al.). Both estrogen and progesterone have been shown to promote type-I interferon and Toll-like receptor pathway immune activation, whilst testosterone enhances T-helper 1 responses. Sex determinants also impact the clinical presentation of autoimmune rheumatic diseases, as males with SLE present more commonly with skin involvement and renal damage (Kim et al.). The impact of endogenous and exogenous sex hormones in RA remains controversial, with the majority of studies investigating only female patients or providing conflicting evidence regarding their impact on RA risk or outcomes, despite the widely recognized anti-inflammatory properties of both androgens and progesterone, and the dichotomous effects of estrogens on immune cell functions (Raine and Giles).

An interesting area of research is the possible adverse pregnancy outcomes associated with autoimmune diseases, such as SLE. Li et al. used transcriptomics to investigate differentially expressed genes (DEGs) in placental tissue from pregnant women with SLE vs. healthy controls, as well as between SLE pregnancies with male or female fetuses. The study identified a SLE disease signature and a SLE pregnancy signature, both disaggregated by the fetal sex, and associated with unique inflammatory pathways, suggesting that fetal-sex-specificity may contribute to the pathophysiology of pregnancy complications in SLE.

Despite the frequent exclusion of the X-chromosome from genome-wide association studies, genetics allows the interrogation of the impact of sex chromosomes on sexual dimorphisms relevant to autoimmunity. Hässler et al. developed a semiparametric additive hazard model accounting for skewed X-inactivation to investigate loci associated with time-to-event data in patients with autoimmune diseases treated with biologic therapies as part of the ABIRISK consortium. Two protective single nucleotide polymorphisms were identified, which can have implications in assessing the sex-biased risk for immunogenicity to biologic treatments.

Autoimmune rheumatic diseases are also characterized by sex bias related to their comorbidity risk. Bruno et al. analyzed data from over 13,000 records of patients with SS and identified that whilst a higher proportion of male patients developed cardiovascular disease (CVD), females frequently had fibromyalgia depression, hypermobility syndromes and migraine, as well as other autoimmune conditions, such as Raynaud's syndrome, SLE and systemic sclerosis.

One of the very few autoimmune rheumatic diseases with male predominance is ANCA (anti-neutrophil cytoplasmic antibody) associated vasculitis (AAV). A meta-analysis found that men with AAV have a 1.54-fold increase of 1-year mortality risk compared to females (Zhu et al.). Additionally, significant sex differences in age of onset, clinical outcome measures, and 1-year mortality rate were observed in a retrospective analysis, highlighting the need for improved sex-tailored early management strategies in AAV.

As the field of sex disaggregated research grows in its prominence, Peckham et al. call for the expansion of such research to include gender-diverse research participants. There is a growing proportion of society frequently excluded from medical and basic research, leading to epidemiological and clinical data that is not necessarily applicable to everyone. By expanding our study designs to include transgender and non-binary people, the authors emphasize the urgent need for long-term outcome data related to gender-affirming hormonal treatments and highlight multiple ways by which future research findings could be improved.

With respect to sex-tailored disease prevention and health care, inflammation and metabolism typically drive autoimmunity and CVD risk bias toward women and men, respectively; however, women with SLE have an increased CVD risk compared to female healthy controls (50-fold). Robinson et al. review this paradox by exploring studies in gender-diverse cohorts and highlighting an estrogen-driven atheroprotective lipoprotein profile in post-pubertal women that is absent in pre-puberty and can be induced by gender-affirming sex hormones in transgender women. Strikingly, this atheroprotective lipid profile is lost in SLE, suggesting a compromise in an autoimmune setting. This highlights potential therapeutic targets to reduce CVD risk in SLE.

Animal models of health and disease have long complemented and enhanced data from human studies. The bidirectional relationship between sex hormones and gut-microbiota—both of which are known to influence autoimmunity development is explored by Rosser et al. in a review which highlights the impact of sex hormones and the potential to modulate the gut-microbiome to influence the course of autoimmune diseases. The observations encourage to consider the therapeutic potential of the complex interplay between the myriad of microbial species that inhabit our bodies and the immune and endocrine systems.

Our understanding of sexual dimorphisms in immune responses and metabolism relevant to autoimmunity has evolved with the accelerated use of complex multi-omic techniques and analysis methods, as well as access to gender-diverse cohorts and sex/gender-specific animal models. However, many questions remain unanswered and future efforts need to account for sex and gender in human and animal research.

## Author contributions

GR, HP, and CC drafted the manuscript. All authors reviewed and approved the editorial.

